# *Campylobacter* populations in wild and domesticated Mallard ducks (*Anas platyrhynchos*)

**DOI:** 10.1111/j.1758-2229.2011.00265.x

**Published:** 2011-10

**Authors:** Frances M Colles, Jan S Ali, Samuel K Sheppard, Noel D McCarthy, Martin C J Maiden

**Affiliations:** The Department of Zoology, University of OxfordSouth Parks Road, Oxford, OX1 3PS, UK

## Abstract

Identifying the *Campylobacter* genotypes that colonize farmed and wild ducks will help to assess the proportion of human disease that is potentially attributable to the consumption of duck meat and environmental exposure to duck faeces. Comparison of temporally and geographically matched farmed and wild ducks showed that they had different *Campylobacter* populations in terms of: (i) prevalence, (ii) *Campylobacter* species and (iii) diversity of genotypes. Furthermore, 92.4% of *Campylobacter* isolates from farmed ducks were sequence types (STs) commonly associated with human disease, in contrast to just one isolate from the wild ducks. Only one ST, ST-45, was shared between the two sources, accounting for 0.9% of wild duck isolates and 5% of farmed duck isolates. These results indicate that domestic ‘niche’ as well as host type may affect the distribution of *Campylobacter*, and that husbandry practises associated with intensive agriculture may be involved in generating a reservoir of human disease associated lineages.

## Introduction

*Campylobacter* continues to be a major cause of bacterial gastroenteritis worldwide, with a reported incidence of 12.79/100 000 population in the USA and 51 488 reported cases in the UK in 2007 (HPA, 2008; Vugia *et al*., 2008). Although the disease is largely sporadic and self-limiting, more serious sequelae such as Guillain–Barrré syndrome and reactive arthritis can occasionally occur and the total annual economic burden has been estimated to be $8 billion in the USA and £500 million in the UK (Buzby and Roberts, 1997; Ketley, 1997; Nachamkin *et al*., 1998; Humphrey *et al*., 2007; Townes *et al*., 2008). In a UK study, 93% of human infection was caused by *Campylobacter jejuni*, with most of the remainder caused by *Campylobacter coli* (Gillespie *et al*., 2002). *Campylobacter* species can be isolated from the intestinal tract of many animals and birds and also from environmental sources such as water.

Approximately, 18 million ducks were produced in the UK in 2006 (Jones *et al*., 2009). While chicken is an important source of human infection, a UK study found 50.7% of duck meat to be contaminated with *Campylobacter*, which is comparable with chicken meat (60.9%), and another attributed 2% of Campylobacteriosis outbreaks to duck meat (Little *et al*., 2008; Sheppard *et al*., 2009). Other studies report duck meat contamination rates of 6–36% (depending on sample type) in Egypt, 31% in Thailand and 45.8% in Ireland (Khalafalla, 1990; Whyte *et al*., 2004; Boonmar *et al*., 2007). Pekin is currently the duck strain most commonly reared for commercial meat production and it originates from domestication of the Mallard (*Anas platyrhynchos*) (Rodenburg *et al*., 2005). Wild ducks also pose a potential health risk to humans, with reported *Campylobacter* carriage rates ranging from 13% to 75%, although models attribute a low proportion of human disease to environmental sources (Luechtefeld *et al*., 1980; Pacha *et al*., 1988; Obiri-Danso and Jones, 1999; Fallacara *et al*., 2001; Wilson *et al*., 2008; Sheppard *et al*., 2009).

The aim of this observational study was to explore the potential of multilocus sequence typing (MLST) in describing and comparing the genetic diversity of *Campylobacter* colonizing domesticated (farmed) and wild Mallard ducks, using isolates from faecal samples. Identifying the genotypes colonizing farmed and wild ducks is necessary to assess the proportion of human disease attributable to consumption of duck meat, as opposed to environmental exposure to duck faeces such as in recreational water. The degree of difference between the populations in these two duck associated sources will determine whether genetic attribution models can differentiate between them. It also allows assessment of the extent to which there are duck associated *Campylobacter* genotypes and whether these are robust to the different ecology of these genetically similar groups of ducks.

## Results and discussion

### *Campylobacter* prevalence

**Fig. 1:**
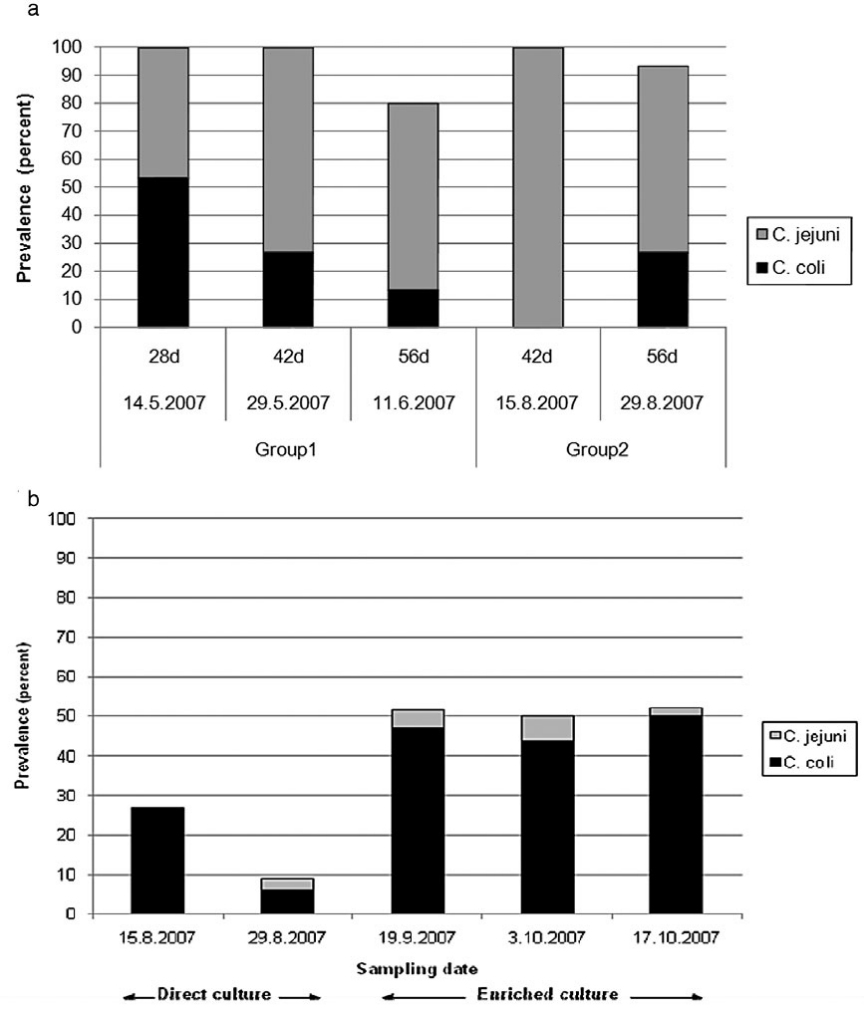
The prevalence and proportion of *Campylobacter* species isolated from (a) two groups of 60 farmed ducks aged 28–56 days and (b) 60–100 wild Mallard ducks on five sampling occasions (August–October 2007). Faecal samples were collected from each of 15 pens containing four domesticated ducks and separated by wooden partitions at the University farm, Wytham (Jones *et al*., 2009). Wild ducks were sampled on a pond and subsidiary of the River Cherwell in the University Parks approximately 5 miles distant. Freshly voided faecal samples of consistent size and appearance were collected only from areas where Mallards had been observed resting immediately before. To minimize the chance of repeated sampling from the same animal, a cross section of each of the areas was sampled, adjacent specimens were avoided and fewer samples were collected than ducks counted on the pond. *Campylobacter* was isolated on mCCDA for direct culture, and Exeter broth and mCCDA for enriched culture, using standard techniques (Colles *et al*., 2009).

The prevalence of *Campylobacter* among two groups of 60 farmed ducks tested at 28–56 days of age was high (93.3–100%), and was similar to that seen in broiler chickens (Fig. 1) (Frost, 2001; Newell and Fearnley, 2003; Colles *et al*., 2008a). There are few other reports of on-farm prevalence of *Campylobacter* among domestically reared duck flocks, but one found rates to vary from 2.5% to 60% at 69–84 days of age and another recorded 100% colonization at 8 days of age (Kasrazadeh and Genigeorgis, 1987; McCrea *et al*., 2006). There was some evidence of a drop in *Campylobacter* colonization, both in prevalence and average numbers of colony-forming units (cfu) estimated by the Most Probable Number method, in the first group of farmed ducks between the ages of 42 (4.1 × 10^6^ cfu g^−1^) and 56 (2.2 × 10^5^ cfu g^−1^) days of age, and in numbers of cfu only (7.1 × 10^5^ cfu g^−1^ at 42 days and 2.7 × 10^5^ cfu g^−1^ at 56 days) in the second group. There was no significant difference in numbers of cfu evident between the two groups of ducks overall (*P* = 0.73). The changes could be a consequence of maturing immunity, seasonal differences or the dynamics of infection associated with the introduction of new *Campylobacter* genotypes (Wallace *et al*., 1997; 1998;).

The prevalence of *Campylobacter* among the wild ducks was much lower (9.2–52.2%). The higher prevalence rates (50.0–52.2%) were recovered using enrichment broth but still below the prevalence in the farmed ducks (Fig. 1). Other studies report carriage rates of 13–75%, with levels lower among ducks feeding largely on vegetation compared with those straining the sediments of ponds (Luechtefeld *et al*., 1980; Pacha *et al*., 1988; Obiri-Danso and Jones, 1999; Fallacara *et al*., 2001). The conditions in which the farmed and wild birds live were of course very different, with *Campylobacter* prevalence among faecal droppings from the wild ducks likely to be influenced by more extreme differences in temperature, moisture and ultra-violet light levels (Obiri-Danso and Jones, 1999).

### *Campylobacter* species and sequence type diversity

The *Campylobacter* species distribution was markedly different, with *C. jejuni* predominant among isolates from farmed ducks (average of 74.6%) and *C. coli* predominant among isolates from wild ducks (average of 85.7% using direct culture and 90.9% using enrichment culture) (Fig. 1). Species distribution differs in other reports, with *C. jejuni* being the principal species isolated in other studies of wild ducks and wild geese in the UK, while either *C. coli* or *C. jejuni* predominate among duck meat products (Obiri-Danso and Jones, 1999; Boonmar *et al*., 2007; Little *et al*., 2008; Colles *et al*., 2008b).

The STs and clonal complexes identified among 92 isolates from farmed ducks and 109 isolates from wild ducks are given in Table 1. Forty-seven STs were recovered from wild ducks, compared with 10 from farmed ducks. The average diversity index, *D*, over five sampling occasions was 0.54 (range 0.15–0.70) for the farmed ducks and 0.93 (range 0.91–0.96) for the wild ducks, with a zero value indicating that that all individuals within a population are identical and a value of one indicating that they are all different (Hunter, 1990). In order to obtain an indication of the diversity of *Campylobacter* carried by an individual bird, within the constraints of the microbiological sampling frame, up to 10 colonies were sequence typed from a small proportion of birds (ten farmed, four wild, on multiple sampling dates). Carriage of multiple STs was lower among farmed ducks (1–3 STs per bird, average *D* = 0.19, range 0–0.56) compared with wild ducks (1–5 STs per bird, average *D* = 0.42, range 0–0.86). Only one ST, the *C. jejuni* ST-45, with fine type *flaA* SVR allele 2, peptide 27, was shared between both host types, accounting for 0.9% of wild duck isolates and 5% of farmed duck isolates (Meinersmann *et al*., 1997; Dingle *et al*., 2002). This genotype is thought to show adaption for environmental survival and is frequently isolated from water (Kwan *et al*., 2008; Sopwith *et al*., 2008; Carter *et al*., 2009).

**Table 1:** The *C. jejuni* and *C. coli* genotypes identified among isolates from temporally and geographically matched wild (*n* = 109) and farmed (*n* = 92) ducks sampled in Oxfordshire, UK

			Frequency		No. of sampling occasions present
				
Species	Clonal complex	ST	Wild	Farmed	
*C. jejuni*	ST-21 CC	19		14	2
	ST-42 CC	42		1	1
		447		12	3
	ST-45 CC	45	1	34	2
	ST-354 CC	354		1	1
	ST-443 CC	51		1	1
	ST-574 CC	574		3	1
	ST-702 CC	702	2		2
	ST-1287 CC	945		7	1
	ST-1332 CC	1276	1		1
	Unassigned	3321	2		1
		2221	1		1
		3322	1		1
		3536	1		1
		3534	1		1
*C. coli*	ST-828CC	827		15	3
		867		4	1
	Unassigned	3311	14		3
		1986	9		3
		3306	9		2
		3309	6		2
		3319	6		1
		1765	4		2
		3304	4		3
		3532	4		2
		1764	3		2
		2015	3		2
		3821	3		1
		1771	2		2
		3305	2		2
		3820	2		1
		3314	2		2
		3312	2		1
		3308	2		2
		1766	1		1
		1992	1		1
		3307	1		1
		3310	1		1
		3313	1		1
		3315	1		1
		3316	1		1
		3317	1		1
		3318	1		1
		3320	1		1
		3323	1		1
		3533	1		1
		3535	1		1
		3822	1		1
		3823	1		1
		3824	1		1
		3825	1		1
		3826	1		1
		3827	1		1
		3828	1		1
		3829	1		1
		3830	1		1

Up to 10 colonies were genotyped from a small proportion of birds (ten farmed, four wild) and those STs isolated multiple times from the same bird are not included, different STs isolated from the same bird are. Multilocus sequence typing (MLST) was performed using standard methods that have been previously published (Dingle *et al*., 2001; Miller *et al*., 2005).

No effects on ST distribution or diversity from wild ducks were observed using direct and enrichment methods in this study, over that which may be explained by natural turnover within the population. Six STs (one *C. jejuni* and five *C. coli*) were recovered at the same low frequency from both direct and enriched culture, while many more were seen on one occasion only, irrespective of the culture method used. Despite concerns that Exeter broth is more selective for *C. jejuni* due to its Polymixin B content, the predominant growth of *C. coli* from the wild ducks suggests that isolation of this species was not prevented in this case (Humphrey, 1989; Rodgers *et al*., 2010).

### Comparison with other host sources

An equal number of *C. jejuni* STs were recovered from both farmed and wild ducks, but the majority (accounting for 92.4% of the isolates) from farmed ducks were those commonly associated with human disease and farm animal sources (http://pubmlst.org/campylobacter/), in contrast to just one such isolate (ST-45) from the wild ducks. Comparisons with large published population datasets indicated *C. jejuni* from farmed ducks showed largest overlap of STs with nationally sampled retail poultry meat (eight STs) (Sheppard *et al*., 2010) and to a lesser extent with free-range poultry previously sampled on the same farm (four STs) (Colles *et al*., 2008a), but no STs were shared with wild geese sampled on the same farm (Colles *et al*., 2008b). Another study similarly found a *C. coli* serotype common to duck and chicken meat (Little *et al*., 2008). Despite these findings, ST-945, accounting for 7.6% of isolates from farmed ducks and also previously isolated from human disease and chicken meat may have some unusual attributes, as it clusters into the ST-1287 clonal complex dominated by isolates from wild birds, most commonly ‘waders’ (http://pubmlst.org/campylobacter/). In contrast to the farmed ducks, the majority of *C. jejuni* STs from wild ducks showed potential host association, with four being unique to this study, and three being isolated from mallards and/or geese in Sweden, Scotland and/or Denmark between 2002 and 2009 (http://pubmlst.org/campylobacter).

**Fig. 2:**
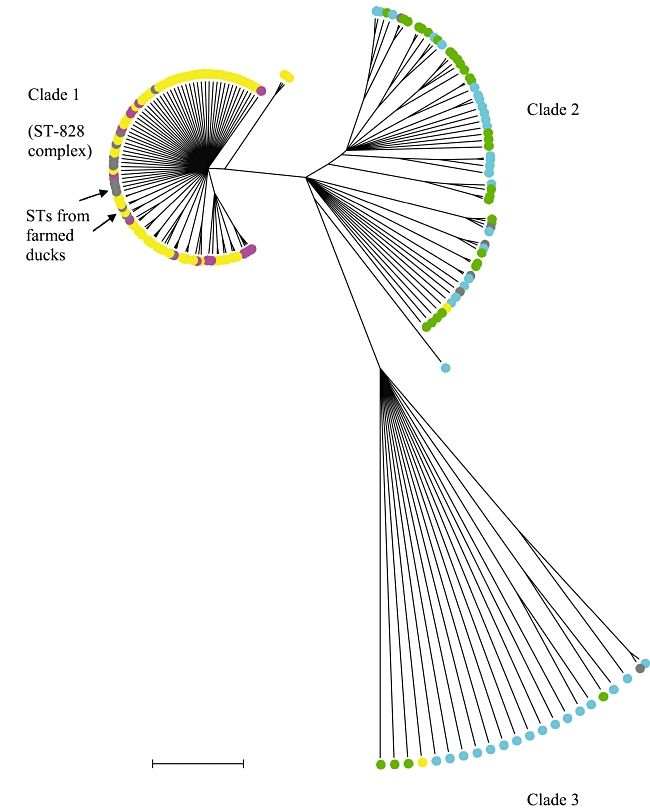
A 75% consensus clonal frame tree demonstrating the genetic relationships between *C. coli* isolates from farmed and wild ducks and other sources, using concatenated nucleotide sequence, 50 000 burn-in cycles and 100 000 further iterations (Didelot and Falush, 2007). Key: environmental water, blue; farmed ducks, highlighted with arrows; wild ducks, green; farmed chicken, pink; retail poultry meat, yellow; shared sources, grey.

The majority (82.1%) of *C. coli* STs from the wild ducks have previously been unreported. Further, *C. coli* STs isolated from the wild ducks frequently contained alleles that were 4–8% divergent from the two *C. coli* STs isolated from farmed ducks and other members of the ST-828 complex predominant in human disease and farm animals, which is supported by the deep branching on the clonal frame tree (Fig. 2) (Didelot and Falush, 2007; Sheppard *et al*., 2008). Unlike *C. jejuni*, clonal complexes have rarely been identified among *C. coli* outside the major ST-828 complex, but seven clusters of two to six closely related STs were identified among the unassigned wild duck isolates and may potentially represent duck or water fowl host associated lineages. Those *C. coli* STs from the wild ducks that were not unique to this study had all previously been isolated solely from water sources. The *C. coli* population from wild ducks exhibited a very high genetic similarity to water isolates, including those sampled as geographically distant as Canada, with very strong support from *F_ST_* analysis demonstrating 96% similarity at the nucleotide level, and a 99% predicted shared ancestry using structure (Wright, 1951; Pritchard *et al*., 2000; Falush *et al*., 2003; McCarthy *et al*., 2007). These isolates from wild ducks and water make up the majority of the two clades that have been recognized in *C. coli* outside of the ST-828 complex (Sheppard *et al*., 2008).

### Concluding remarks and future perspectives

There is increasing evidence of strong host association among *Campylobacter* genotypes (McCarthy *et al*., 2007; Colles *et al*., 2008b; French *et al*., 2009; Sheppard *et al*., 2010); however, the results presented here give a preliminary indication that agricultural practices may alter the microbiota within a given host species, and/or provide an environment by which certain *Campylobacter* genotypes are favoured. It is possible that in developing the Pekin strain over many generations, the caecal function has become sufficiently different to the wild type that there are still affects of host association with the farm bred duck (Clench and Mathias, 1995). Alternatively, the farm environment leads to major differences in diet, age and population structure, immune function, stocking density and behaviours compared with that experienced by wild birds, all of which may affect *Campylobacter* prevalence and diversity. Transmission may be interrupted among wild birds through greater mixing of diverse individuals, but enhanced by rapid replenishment of relatively immature ducks in the domestic setting, potentially favouring bacterial flora adapted to such different dynamics. Large-scale sampling of environmental isolates such as these are essential in determining the population structure and natural ecology of *Campylobacter*, particularly *C. coli*, more fully. The results from this study are compatible with duck meat being a potential source of human infection and demonstrate the need for large-scale studies across the duck and other non-chicken poultry industries.
